# Bat community assembly in the ecotone of two neotropical biodiversity hotspots is determined by environmental filtering rather than historical factors

**DOI:** 10.1111/1365-2656.70327

**Published:** 2026-08-02

**Authors:** Daiana Cardoso Silva, Hernani F. M. Oliveira, Fabricius M. C. B. Domingos

**Affiliations:** ^1^ Programa de Pós‐Graduação em Ecologia e Conservação Universidade do Estado de Mato Grosso Nova Xavantina MT Brazil; ^2^ Empresa Brasileira de Pesquisa Agropecuária (Embrapa) Porto Velho RO Brazil; ^3^ Departamento de Zoologia Universidade Federal do Paraná Curitiba PR Brazil

**Keywords:** biogeography, CAMI, Chiroptera, ecotone, phylogenetic beta diversity, turnover

## Abstract

1. Ecotones between major biomes represent strong ecological gradients and offer valuable opportunities to investigate the processes structuring biological communities. The ecotone between the Cerrado and the Atlantic Forest is one of the most extensive and biodiverse transition zones in South America.
2. Here, we investigated the assembly processes of bat communities across the Cerrado–Atlantic Forest ecotone, contrasting the predictions of environmental filtering, competitive exclusion, and neutral assembly using a coevolutionary community assembly framework.
3. We also evaluated how environmental and historical variables influence patterns of phylogenetic beta diversity and tested whether abrupt changes in community composition occur along the gradient from the ecotone towards the core areas of both biomes.
4. We found that environmental filtering was better supported than competitive exclusion and neutral models in structuring bat communities within the ecotone.
5. Variation in phylogenetic beta diversity was primarily explained by environmental variables, particularly precipitation seasonality, annual precipitation, annual temperature range, and canopy height.
6. An abrupt change in phylogenetic beta diversity was detected from the ecotone towards the core of the Cerrado biome, whereas no comparable shift was observed towards the Atlantic Forest, indicating that distinct assembly processes operate along gradients associated with these two megadiverse biomes.
7. Our results highlight the dominant role of environmental filtering in shaping bat communities in the Cerrado–Atlantic Forest ecotone and emphasise the importance of accounting for coevolutionary processes when interpreting community assembly patterns in Neotropical biodiversity hotspots.

## Graphical abstract

We demonstrate that environmental filtering is the primary mechanism shaping bat communities across the Cerrado–Atlantic Forest ecotone. By integrating phylogenetic diversity, environmental gradients, and coevolutionary assembly models, our study provides new insights into the processes maintaining biodiversity in a globally important transition zone.

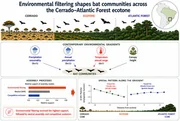

## RESUMO

1. Ecótonos entre biomas com estruturas vegetacionais distintas representam fortes gradientes ecológicos e oferecem oportunidades valiosas para investigar os processos que estruturam as comunidades biológicas. O ecótono entre o Cerrado e a Mata Atlântica é uma das zonas de transição mais extensas e biodiversas da América do Sul.
2. Neste estudo, investigamos os processos de montagem de comunidades de morcegos ao longo do ecótono Cerrado–Mata Atlântica, contrastando as predições dos modelos de filtragem ambiental, exclusão competitiva e montagem neutra por meio de uma abordagem coevolutiva de montagem de comunidades.
3. Também avaliamos como variáveis ambientais e históricas influenciam os padrões de diversidade beta filogenética e testamos se ocorrem mudanças abruptas na composição das comunidades ao longo do gradiente que se estende do ecótono em direção às áreas centrais de ambos os biomas.
4. Verificamos que a filtragem ambiental recebeu maior suporte do que os modelos de exclusão competitiva e de montagem neutra na estruturação das comunidades de morcegos do ecótono.
5. A variação da diversidade beta filogenética foi explicada principalmente por variáveis ambientais, especialmente pela sazonalidade da precipitação, precipitação anual, amplitude térmica anual e altura do dossel.
6. Detectamos uma mudança abrupta na diversidade beta filogenética do ecótono em direção ao núcleo do bioma Cerrado, mas não observamos nenhuma mudança comparável em direção à Mata Atlântica, indicando que diferentes processos de montagem atuam ao longo dos gradientes associados a esses dois biomas megadiversos.
7. Nossos resultados destacam o papel predominante da filtragem ambiental na estruturação das comunidades de morcegos no ecótono Cerrado–Mata Atlântica e enfatizam a importância de considerar processos coevolutivos na interpretação dos padrões de montagem de comunidades em hotspots neotropicais de biodiversidade.

## 1 INTRODUCTION

The spatial distribution of biodiversity and the composition of biological communities are influenced by many abiotic factors (Domingos et al., 2025; Whittaker, 1962). One prominent hypothesis for large‐scale distribution patterns is that species richness in most taxonomic groups varies along a latitudinal gradient, driven by both evolutionary and contemporary ecological processes (Graham et al., 2006). Additionally, ecotone zones between different ecosystems (hereafter referred to as ecotones) are noted for their high biodiversity, likely due to abiotic factors that facilitate the dispersal and establishment of diverse species (Risser, 1995). However, substantial evidence links environmental predictors to large‐scale patterns of ecotone diversity across taxa (Van Rensburg et al., 2009).

Measures of phylogenetic diversity and dispersion, such as overdispersion or clustering, are extensively used in community ecology to infer community assembly processes (Webb et al., 2002). When species within a community are phylogenetically clustered, they tend to be ecologically similar, suggesting that the community may be shaped by environmental or habitat filters that allow species with specific functional traits to thrive (Mayfield & Levine, 2010). Conversely, if species in a community are phylogenetically overdispersed, or less closely related than expected by chance, it implies that competition may have structured the community by excluding species with very similar phenotypes (Cadotte & Davies, 2010; Mayfield & Levine, 2010). Moreover, the spatial variation in phylogenetic structure among communities at a regional scale provides insights into these patterns (Graham & Fine, 2008). These phylobetadiversity metrics enhance our understanding of the factors shaping the structure and composition of ecological communities across environmental gradients, offering valuable perspectives on species interactions and their responses to environmental changes (Silva‐Pereira et al., 2020).

Ecotones feature important ecological gradients that can be studied more effectively using a phylogenetic approach, thereby elucidating historical and contemporary influences on community structure (Kark & Van Rensburg, 2006). These areas are often considered evolutionary cradles (Smith et al., 1997) and offer an important opportunity for robust tests of biogeographic patterns, given their high habitat heterogeneity and distinct climatic conditions, which can be analysed at manageable spatial scales (Moncrieff et al., 2016). On a continental scale, ecotones generally exhibit high species diversity because they encompass species from diverse environments (Risser, 1995). Moreover, because vegetation significantly influences environmental structure, evidence suggests that ecosystem properties and animal communities respond in parallel to variations in vegetation structure (Kark, 2013; Risser, 1995; Van Rensburg et al., 2009).

In this study, we investigated contemporary and historical factors that explain the variation in diversity and phylogenetic structure within the ecotone between two major biodiversity hotspots in South America: the Atlantic Forest and the Cerrado (Myers et al., 2000). The gradual vegetation change in the Cerrado–Atlantic Forest ecotone is primarily influenced by rainfall seasonality and soil moisture, leading to a mix of Cerrado and forest species that makes the vegetation locally homogeneous (Durigan & Ratter, 2006). Historical climatic fluctuations during the Quaternary certainly played a role in the local biodiversity distribution, causing savannah vegetation to expand into previously forested areas during glacial cycles, and vice versa (Durigan & Ratter, 2006). Hence, both contemporary ecological and historical evolutionary processes likely play important roles in shaping current ecotone biodiversity patterns in the region.

Despite hosting an exceptionally high number of species, biodiversity patterns in the Cerrado–Atlantic Forest ecotone have been rarely explored. The bat fauna in these biomes collectively represents approximately 60% of the bat species recorded in Brazil and 8% of the bat species worldwide (~1500 spp.), including threatened and geographically restricted species (Garbino et al., 2024). Environmental predictors, such as altitude and rainfall, are often reported as limiting factors in bat distribution patterns (Farneda et al., 2015; Mendes et al., 2017). This relationship with environmental characteristics suggests that bats are primarily phylogenetically clustered within distinct ecosystems (Grimshaw & Higgins, 2017; Presley et al., 2018). To understand how climate and vegetation structure (as proxies for contemporary factors) and climate stability and elevation (as proxies for historical factors) explain variations in the diversity (phylobetadiversity) and phylogenetic structure (overdispersed or clustered) of bats in the Cerrado–Atlantic Forest ecotone, we investigated the following questions and hypotheses: (i) which ecological processes best explain the assembly of bat communities in the Cerrado–Atlantic Forest ecotone? We hypothesise that ecotone communities will integrate species from different biomes. Given the high habitat heterogeneity and varied climatic conditions, species with similar functional traits will likely compete for resources, leading to competitive exclusion shaping the communities (Mayfield & Levine, 2010; Risser, 1995). (ii) Are the mechanisms that determine community structure in the central regions the same as those in the ecotone between the two biomes? Our hypothesis is that contemporary factors will predominantly determine the structure of bat communities in the ecotone, because of their role in maintaining these environmental gradients (Durigan & Ratter, 2006). Conversely, historical factors will be more influential in the central regions of the biomes, where greater climatic stability can support higher levels of taxonomic and phylogenetic diversity (Costa et al., 2018). (iii) Is the variation in the phylogenetic structure of communities (phylobetadiversity) influenced by the distance from the central regions of the biome to the ecotone? Our hypothesis is that the patterns of phylobetadiversity will vary with distance, depicting species adaptation to the characteristics of each environmental gradient (Risser, 1995; Toms & Lesperance, 2003). This will result in points of interruption in phylobetadiversity, with abrupt compositional changes between the ecotone and central regions of the biomes.

## 2 MATERIALS AND METHODS

### 2.1 Biomes overview

We conducted our analyses in the biogeographic ecotone between two biodiversity conservation hotspots in Brazil: The Cerrado and the Atlantic Forest. The Cerrado, the second‐largest Neotropical biome, is considered the richest savannah in the world (Klink & Machado, 2005). Extending approximately 26° of latitude (2°N–24°S) across central Brazil, this biome comprises a mosaic of dry forests, seasonally dry tropical forests, and dense, open, and flooded savannas (Ribeiro & Walter, 2008). The Atlantic Forest is located along the east coast of South America, spanning an extensive area along a latitudinal gradient of about 30° (3°S–33°S) (Ribeiro et al., 2009). This biome includes a variety of vegetation formations, such as restingas, coastal forests, dense forests, mixed forests, and semi‐deciduous forests (Oliveira‐Filho & Fontes, 2000).

### 2.2 Species data

We compiled a database of bat communities in the Cerrado and Atlantic Forest biomes by combining occurrence data from two existing databases: one in the Cerrado (Silva et al., 2024) and one in the Atlantic Forest (Aguiar et al., 2020; Muylaert et al., 2017). Each sampling unit consisted of a list of all species present in each community. We standardised the Atlantic Forest data using a grid of approximately 20 km and extracted presence–absence information for each grid cell, consistent with the methodology used for the Cerrado communities (Silva et al., 2024). We defined the geographic boundary of the Cerrado–Atlantic Forest ecotone based on studies that used climatic and soil predictors to establish biome limits and emphasised their ecological and biogeographic relevance (Arruda et al., 2024; Souza et al., 2024). This allowed us to clearly identify bat communities within the ecotone region, the Cerrado and the Atlantic Forest. To minimise the influence of other biogeographic factors shaping community assembly in the two biomes, we excluded communities near other surrounding biomes (Caatinga, Amazonia, and Pantanal) considering 100 km to the boundaries of such biomes. Additionally, we excluded communities with fewer than six species to reduce sampling bias associated with variation in sampling effort and inventory completeness across the different data sources used in this study (Silva et al., 2024; Zortéa & Alho, 2008). This approach, and all our analyses considering standardised effect sizes (SESs) (Section 2.5), provide a framework in which communities are comparable across the geographic scale of our study (Aguiar et al., 2020; Delgado‐Jaramillo et al., 2020; Silva et al., 2022, 2024). Our analysis included a total of 225 communities comprising 118 bat species: 84 communities in the Cerrado, 117 in the Atlantic Forest, and 24 in the ecotone (Figure 1). This study was based exclusively on previously published occurrence records and trait data compiled from the literature and public repositories, and did not involve direct animal handling, capture, or field sampling. Therefore, ethical approval and fieldwork permits were not required.

**FIGURE 1:**
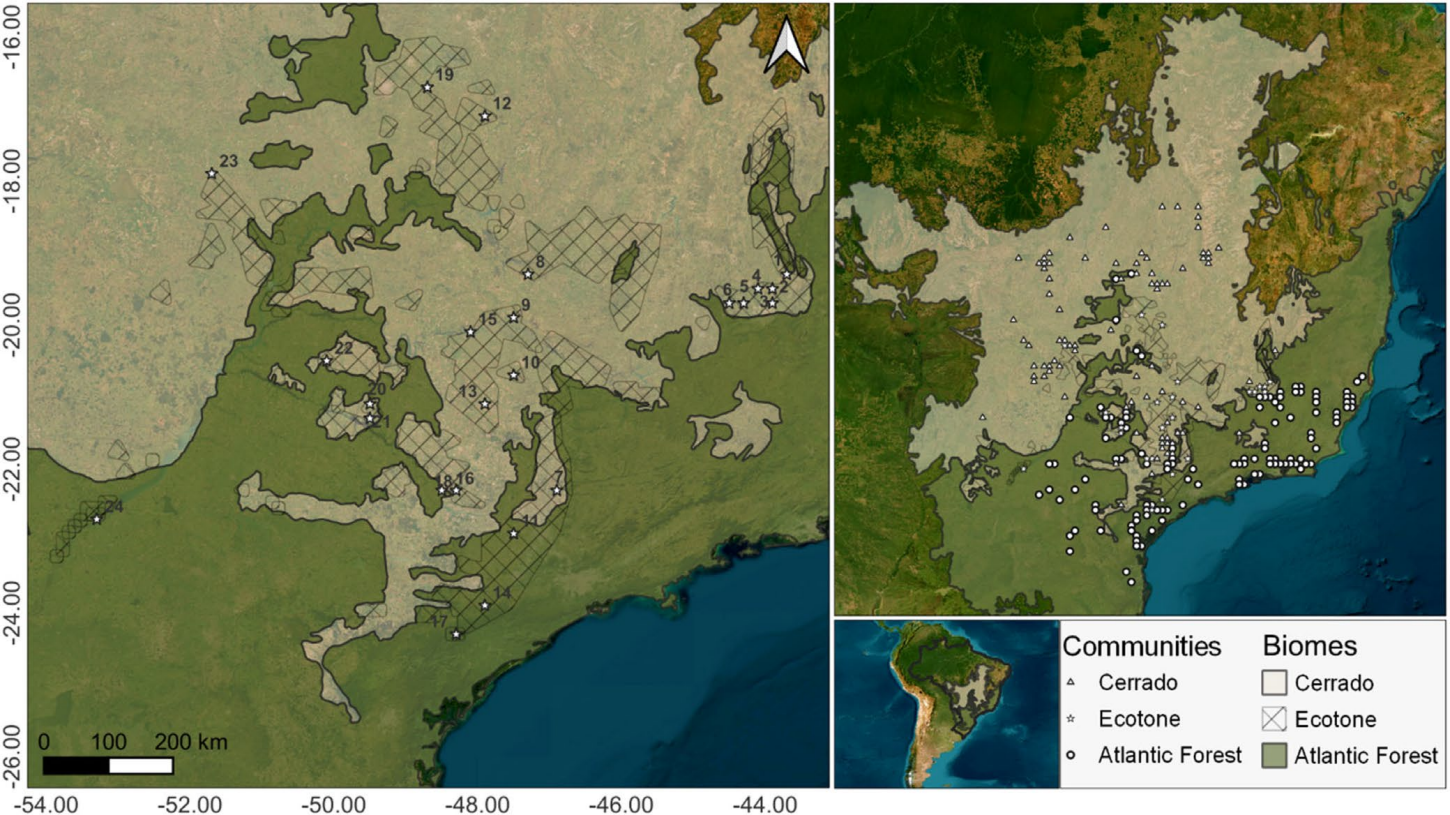
Distribution of bat communities in the Cerrado and Atlantic Forest biomes, and the ecotone between these two biodiversity hot spots. On the left, the distribution of the 24 bat communities in the Cerrado–Atlantic Forest ecotone is shown, with numerical identification from 1 to 24. On the right, the distribution of all communities used in this study, showing the 84 communities in the Cerrado (triangles), 117 in the Atlantic Forest (circles), and 24 in the ecotone (stars).

### 2.3 Phylogenetic and functional data

We used the most recent and comprehensive bat phylogeny with species‐level resolution (Upham et al., 2019, available at VertLife). To estimate functional diversity and infer community assembly processes, we compiled four functional traits from the literature (Aguiar et al., 2016; Carvalho et al., 2019; Kissling et al., 2014; Marinello & Bernard, 2014; Norberg & Rayner, 1987; Silva et al., 2024). Most trait data used in this study were obtained from the comprehensive compilation published by Silva et al. (2024), which also provides the original references for each species (see Table S1.3 for more details). When only relative wing loading (RWL) values were available, they were converted to wing loading (WL) following Norberg and Rayner (1987). Because WL and aspect ratio (AR) data were unavailable for 14 of the 118 species included in the analyses, these values were estimated using phylogenetic generalised least squares (PGLS) models based on forearm length and phylogenetic relationships among species (Silva et al., 2024). All selected traits are related to bat wing and body size, representing different habitat use particularities. *WL* is an important proxy for bat flight capacity and resource partitioning (Luo et al., 2019; Marinello & Bernard, 2014; Norberg & Rayner, 1987). *AR* is defined as the relationship between a bat's wingspan and its body size, influencing flight efficiency and speed (Norberg & Rayner, 1987). *Forearm length* is highly correlated with body size, making it an informative and simple‐to‐measure trait (Castillo‐Figueroa & Pérez‐Torres, 2021). *Body mass* is related to body size and influences many functions and behaviours, such as wingbeat frequency, bite force, and basal metabolic rate (Castillo‐Figueroa & Pérez‐Torres, 2021). We used these morphometric traits because they are fundamental for assessing the ecological fit of bats to different environments and, as such, are optimal traits for inferring which ecological processes dominate in bat communities.

### 2.4 Environmental predictors

We tested the influence of ecological determinants using 19 bioclimatic variables, which are available in the WorldClim v2 environmental dataset (Fick & Hijmans, 2017; http://www.worldclim.org) with a resolution of 0.01° (~1 km). To reduce the dimensionality of these variables and avoid multicollinearity, we used the variance inflation factor (VIF) with the *usdm* package (Naimi, 2017), setting a cut‐off value of VIF >3 to indicate high collinearity, retaining only variables with VIF ≤3 (Zuur et al., 2010) in the following analyses. We calculated the VIF for each environment separately: Cerrado, Atlantic Forest, and Ecotone (see Appendix S1.1).

We used two variables to represent vegetation structure: (1) we calculated the proportion of natural vegetation cover surrounding each community using land‐cover data from the MapBiomas 8.0 collection (https://mapbiomas.org/colecoes‐mapbiomas‐1?cama set language = en‐BR) with a time series of 15 years with a 2‐year interval between them (2005–2019), and manipulated it in Google Earth Engine. We used a spatial resolution of approximately 30 m and created an approximately 20 km buffer around each community centroid. Thus, we extracted the proportion of natural vegetation with eight layers between 2005and 2019 and, finally, we calculated the average of this time series. In addition (see Appendix S2.1), (2), we calculated the average canopy height (Simard et al., 2011) with a *buffer* of approximately 20 km around each centroid, and we extracted the information with 576 points distributed in each community (cell) using the same spatial resolution as the original layer (~1 km^2^).

We tested the influence of historical factors using digital elevation data from the *Shuttle Radar Topography Mission* (SRTM‐https://www.earthdata.nasa.gov/data/instruments/srtm) with a spatial resolution of one arcsecond (~30 m) using the *raster* package (Hijmans et al., 2020). We also used two alternative climatic stability proxies: climate stability data based on floristic change in South America over the last 30,000 years (Costa et al., 2018) (hereinafter, instability) and the continuous estimate of climate stability from 21,000 years ago from the StableClim dataset, based on the TraCe21ka experiment (Fordham et al., 2017) (hereinafter, paleoclimate). This latest model reconstructs important regional palaeoclimate fluctuations during the last deglaciation event and accurately simulates current climate patterns (Fordham et al., 2017).

We calculated the distance (km) in the QGIS environment (https://qgis.org) among the centroids of each Cerrado and Atlantic Forest community to the closest point in the ecotone region to check for abrupt changes in phylobetadiversity. We standardised all independent variables (standard deviation = 1 and mean = 0) before using them in subsequent statistical analyses.

### 2.5 Data analyses

To answer our first question, we used the Community Assembly Model Inference (CAMI) analysis (Ruffley et al., 2019). This approach evaluates how communities are assembled by comparing observed phylogenetic patterns with mathematical models representing alternative assembly processes, including neutral assembly, environmental filtering, and competitive exclusion. These processes generate distinct phylogenetic patterns, ranging from clustering to overdispersion, allowing inference on the mechanisms underlying community assembly. This analysis can be summarised in four stages: (1) definition of the regional communities (Cerrado and Atlantic Forest), which represent the regional species pools, and the local communities located within the ecotone region, which were assembled from these pools and for which the assembly models were inferred; (2) simulation of trait evolution under the Brownian motion (BM) model (Felsenstein, 1985) or Ornstein–Uhlenbeck (OU) model (Butler & King, 2004). For this stage, we carried out a principal component analysis (PCA) using the four functional traits (wing loading, aspect ratio, forearm length, and body mass). Because the model allows for only a single trait to be included in the analysis, we retained the first principal component, which explained 63% of the total variance in the dataset. We used the OU model because it performed better in our analysis (see Appendix S1.2); (3) simulation with 1000 repetitions using the phylogenetic data representing the regional species pool, and phylogeny of local (ecotone) species with the trait evolution model (OU) for each assembly model, that is, neutral, environmental filter and competitive exclusion; and, (4) estimating model performance using *Random Forest* (RF) and *Approximate Bayesian Computation* (ABC). At the end, we can assess which of the three models (neutral, environmental filter, and competitive exclusion) best explain the probability of species in the regional pool (Cerrado and Atlantic Forest) to be present in the communities sampled in local communities of interest, in this case, the Cerrado–Atlantic Forest ecotone. The neutral model is the one in which species from regional communities (Cerrado and Atlantic Forest) have the same probability of persisting in local communities (ecotone) (Hubbell, 2001). The environmental filter model is selected by a coevolutionary approach that correlates traits among species and their environment with the probability of persistence in a community (details in Ruffley et al., 2019).

To test hypotheses 2 and 3, we calculated phylogenetic beta diversity (PBD) (βSOR) and its turnover (βSIM) and nestedness (βSNE) components based on phylogenetic diversity (Faith's PD) using Sørensen's index with the phylo.beta.pair function from the *betapart* package (Baselga & Orme, 2012), which is based on the total length of shared and unshared branches between paired communities. We used null models to compare observed and random species assemblages to assess differences in PBD (Molina‐Venegas, 2019). We generated null communities using the *phylosor.rnd* function in the *picante* package (Kembel et al., 2010), randomising the terminals in the phylogeny 999 times while keeping the total number of species constant (Leprieur et al., 2012). We then calculated the SES to test whether our observed PBD differs from the random expectation. For this step, we used the ses.PBD function (https://pedrohbraga.github.io/CommunityPhylogenetics‐Workshop/CommunityPhylogenetics‐Workshop.html), with

$$\mathrm{SES}=\frac{\text{Xobs}-\text{Xnull}}{\text{SDXnull}}$$

where *X*obs is the observed PhyloSor value, Xnull is the mean of the null distribution, and s.d. (Xnull) is the standard deviation of the null distribution (Leprieur et al., 2012). Thus, we can determine whether the communities are more different than expected by chance (i.e. obs > mean(null) = SES.PhyloSor greater than 1.96); more similar than expected by chance (i.e. obs < mean(null) = SES.PhyloSor less than −1.96); or randomly dissimilar, that is, as different as we would expect by chance (obs = mean(null) = 0 PhyloSor). We will use the acronym PBD (*phylogenetic beta diversity*) to refer to the observed PBD, and SES.PBD to the SES.

To answer the second question and choose the best predictors of PBD and SES.PBD, we used machine learning methods based on RF algorithms with the VSURF package (Genuer et al., 2015). In this approach, the selection of variables is carried out in three stages: the first stage is preliminary variable selection and classification, that is, it classifies the variables of importance (VI) and eliminates redundant and irrelevant variables; the second stage selects the variables involved in the RF model that have the lowest error values (*out‐of‐bag error*, OOB); in the third stage, a variable inclusion threshold is set based on the average of the absolute OOB values before the exclusion of less important variables (Genuer et al., 2015).

To verify the geographical variation of PBD and SES.PBD in the environmental gradient, and answer our third question, we performed a piecewise regression, a statistical technique used to model ecological thresholds (Toms & Lesperance, 2003). We chose the *segmented* package (Muggeo, 2008) to identify breakpoints in the values of *x* (distance) where the slope of the linear function changes.

## 3 RESULTS

### 3.1 Overall trends

Of the 225 communities with 118 bat species, 24 were located in the ecotone, with a total of 62 bat species. The species in the ecotone belong to seven families of Chiroptera: Phyllostomidae (35 species), Molossidae (12 species), Vespertilionidae (10 species), Noctilionidae (2 species), and Emballonuridae, Furipteridae, and Natalidae (one species each) (see Appendix S1.3). Regarding co‐occurrence, 58 species occur in the three environments, Ecotone, Cerrado, and Atlantic Forest; three species occur in the Ecotone and Atlantic Forest; and one species occurs in Ecotone and Cerrado.

In the Cerrado–Atlantic Forest ecotone (24 communities), total PBD was β_PBD = 0.44 ± 0.04 (Figure 2a). This pattern was driven primarily by the turnover component (β_SIM = 0.27 ± 0.05; Figure 2c), with a smaller contribution of nestedness (β_SNE = 0.17 ± 0.05; Figure 2b). The Cerrado and Atlantic Forest biomes followed the same pattern (β‐total = 0.48 ± 0.07 and 0.47 ± 0.06, respectively) (Figure 2a). The variation in SES.PBD among the ecotone, Cerrado, and the Atlantic Forest is similar (SES = −0.84 ± 0.40, −0.98 ± 0.66 and −0.78 ± 0.50, respectively) (Figure 2d). There were no significant SES.PBD values for the ecotone and Atlantic Forest regions, whereas in the Cerrado six communities had significant values below −1.96. This result indicates that most of the communities can be explained by the observed PBD.

**FIGURE 2:**
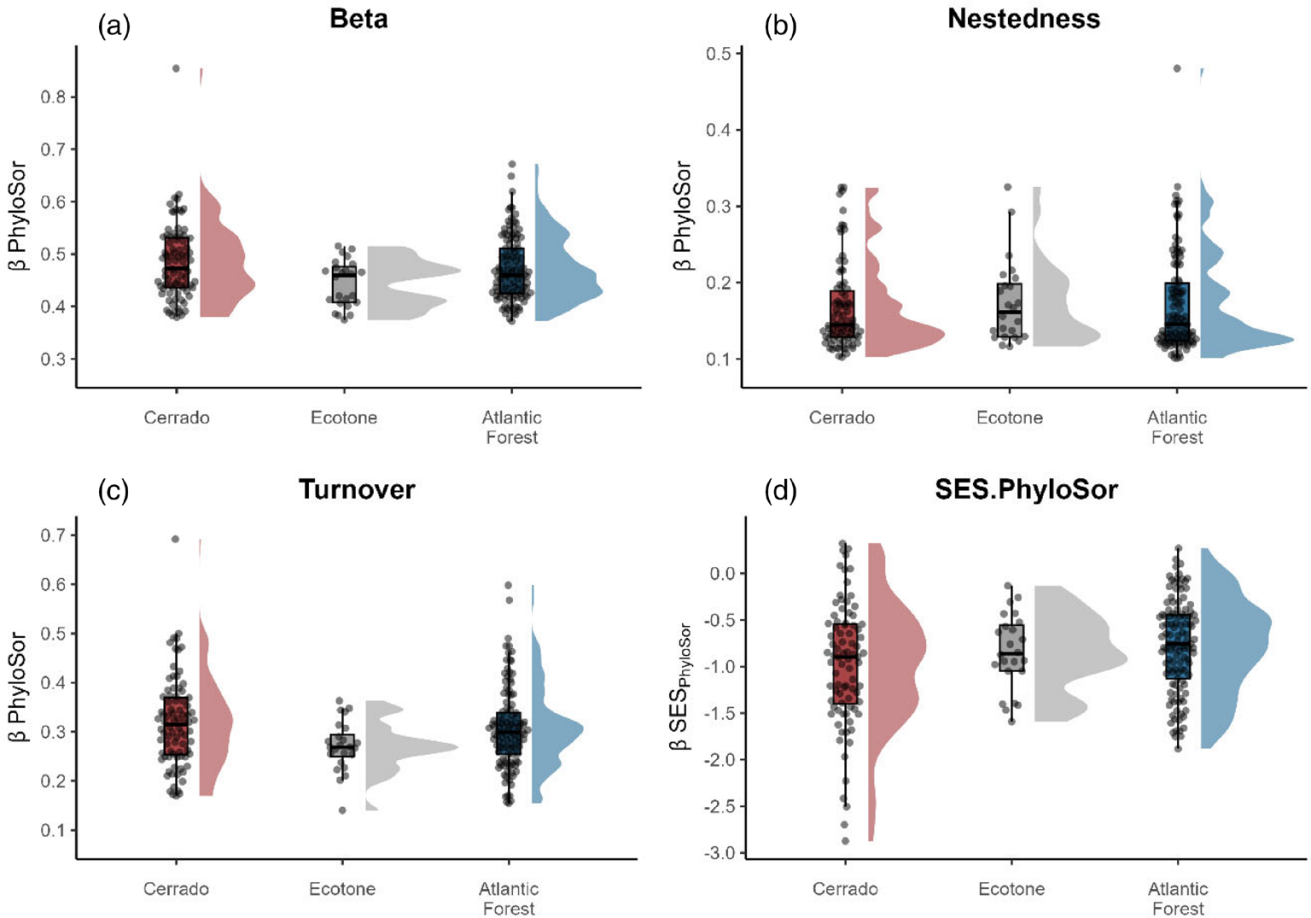
Observed patterns of phylobetadiversity in bat communities from the Cerrado–Atlantic Forest ecotone. (a) Observed phylobetadiversity calculated by Sorensen's index with partitions turnover and nestedness for each studied region; (b) Phylobetadiversity calculated by Sorensen's index with a standardised effect that keeps species richness constant in each community.

### 3.2 Community assembly process

Contrary to our first hypothesis, the predictions of the community assembly process using the RF and ABC model posterior probabilities for the 24 communities in the ecotone indicated the highest support for the environmental filtering model, with the second highest support for the neutral model and little support for exclusive competition (Figure 3, Table S1.4). The OOB error rates for the RF classifier were 28.8% (0.288) (see Appendix S2.2). The parameter estimates $\;t_{E}$ for the environmental filter model showed intermediate values (close to 30) for each community in an OU model of trait evolution (Figure S2.3, Table S1.5). The RF and ABC showed an important relationship between the strength of the environmental filtering process and the support for the best model (Table S1.5, Figure S2.4).

**FIGURE 3:**
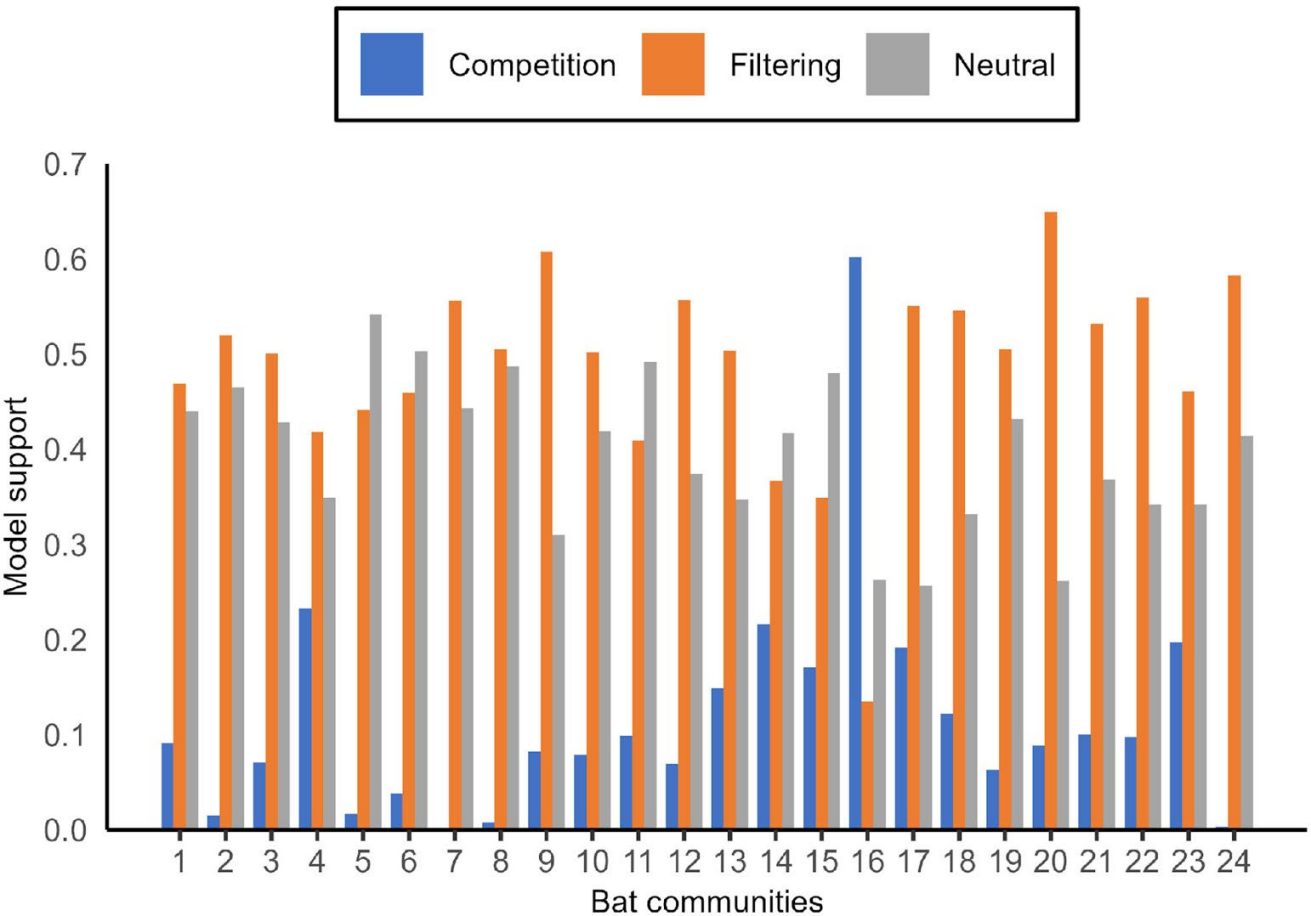
Assembly model predictions for the 24 bat communities in the Cerrado–Atlantic Forest ecotone. Model support estimated by the *Random Forest* algorithm.

### 3.3 Main predictors of phylogenetic beta diversity

The variables selected using our *RF* approach corroborated our hypothesis that contemporary factors determine the variation of PBD and SES. PBD in the ecotone region (Table 1). The SES.PBD of the ecotone was best explained by precipitation seasonality (bio15), annual precipitation (bio12), and temperature annual range (bio7) (Figure 4a–c). The PBD in the ecotone was best explained by canopy height (Figure 4d). The PBD and SES.PBD of the Cerrado and Atlantic Forest biomes were explained by both contemporary and historical factors (Table 1 and Appendices S2.5 and S2.6). The SES.PBD of the Cerrado was explained by precipitation of the coldest quarter (bio19), precipitation of the warmest quarter (bio18), and canopy height. The PBD of the Cerrado was influenced by precipitation in the warmest and coldest quarters (bio18 and bio19), mean temperature of the wettest quarter (bio8), and palaeoclimate. The SES.PBD of the Atlantic Forest was explained by elevation and palaeoclimate. PBD was best explained by elevation, canopy height, annual temperature range (bio7), and palaeoclimate.

**TABLE 1:** Importance scores of the variables generated by the *Random Forest* models for phylogenetic beta diversity (PBD) and standardised effect size of phylogenetic beta diversity (SES.PBD) of bat communities from the Cerrado, Atlantic Forest and ecotone. In bold are the significant variables for each biome and the ecotone.

Variables	SES.PBD			PBD		
	Ecotone	Cerrado	Atlantic forest	Ecotone	Cerrado	Atlantic forest
Paleoclimate	−0.0048	0.0079	**0.0297**	−0.0001	**0.0002**	**0.0004**
Instability	−0.00106	−0.0013	0.0265	−0.0001	0.0000	0.0003
Vegetation	−0.0032	0.0143	0.0155	−0.0001	0.0000	0.0001
Vertical stratification	−0.0063	**0.0226**	0.0034	**0.0001**	0.0000	**0.0004**
Elevation	0.0003	0.0129	**0.0572**	−0.0001	0.0000	**0.0005**
Annual precipitation (Bio12)	**0.0122**	0.0074	0.0067	−0.0001	0.0001	0.0001
Precipitation seasonality (Bio15)	**0.0128**	—	—	−0.0001	—	—
Precipitation of warmest quarter (Bio18)	−0.0025	**0.0238**	—	**−**0.0001	**0.0004**	—
Temperature annual range (BIO5‐BIO6) (Bio7)	**0.0060**	0.0086	0.0152	−0.0001	0.0001	**0.0004**
Mean Temperature of Wettest Quarter (Bio8)	−0.0037	0.0138	0.0216	−0.0001	**0.0003**	0.0003
Precipitation of coldest quarter (Bio19)	—	**0.0248**	—	—	**0.0003**	—
Precipitation of wettest month (Bio13)	—	—	0.0050	—	—	0.0001
Isothermality (BIO2/BIO7) (×100) (Bio3)	—	0.0030	0.0199	—	0.0000	0.0002

**FIGURE 4:**
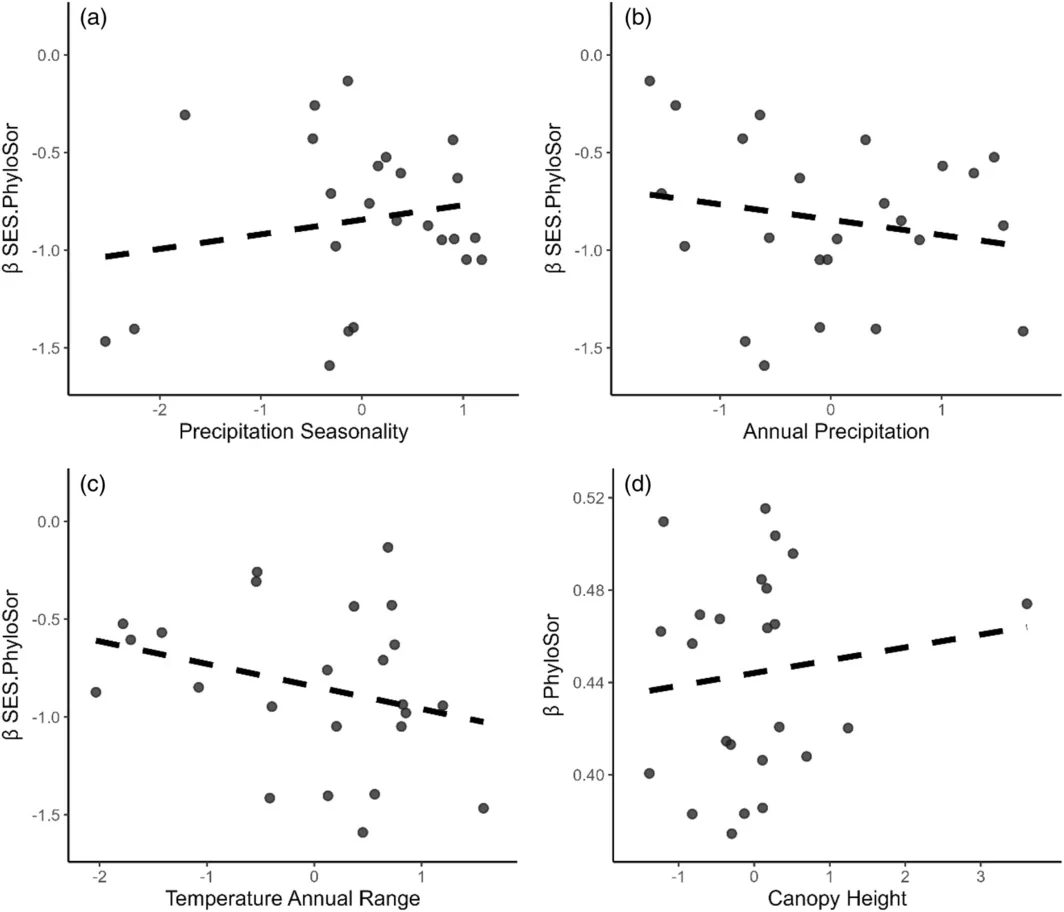
Simple regression to help visualise the general relationship trends between environmental variables and phylobetadiversity metrics of bat communities from the Cerrado–Atlantic Forest ecotone. Importance of variables chosen for representation was estimated by *Random Forest*. Standirdised phylogenetic beta diversity (SES.PhyloSor) in relation to precipitation seasonality (a), annual precipitation (b), and temperature annual range (c). Observed phylogenetic beta diversity (PhyloSor) in relation to mean canopy height (d).

### 3.4 Variation in phylobetadiversity with distance

The relationship between the distance from the ecotone region to the estimated breakpoints in the variation of SES.PBD and PBD were significant for the Cerrado biome (*r* ^2^ = 0.11, *p* < 0.01 and *r* ^2^ = 0.27, *p* < 0.00001, respectively). Specifically, we found a breakpoint at 153 km, indicating an abrupt change in the variation of SES.PBD, that is, there is an increase in SES.PBD from the ecotone region towards the more central regions of the Cerrado biome (Figure 5a). On the other hand, the variation in PBD has an inverted slope, and the breakpoint that occurs at 300 km has an abrupt change with a negative relationship, that is, there is a decline in PBD towards the central regions of the Cerrado (Figure 5b). The relationship between distance from the ecotone region to estimate breakpoints or changes in the variation of SES.PBD and PBD were not significant for the Atlantic Forest (*p* > 0.05), that is, distance from the ecotone region does not explain compositional breakpoints in the variation of communities in this region.

**FIGURE 5:**
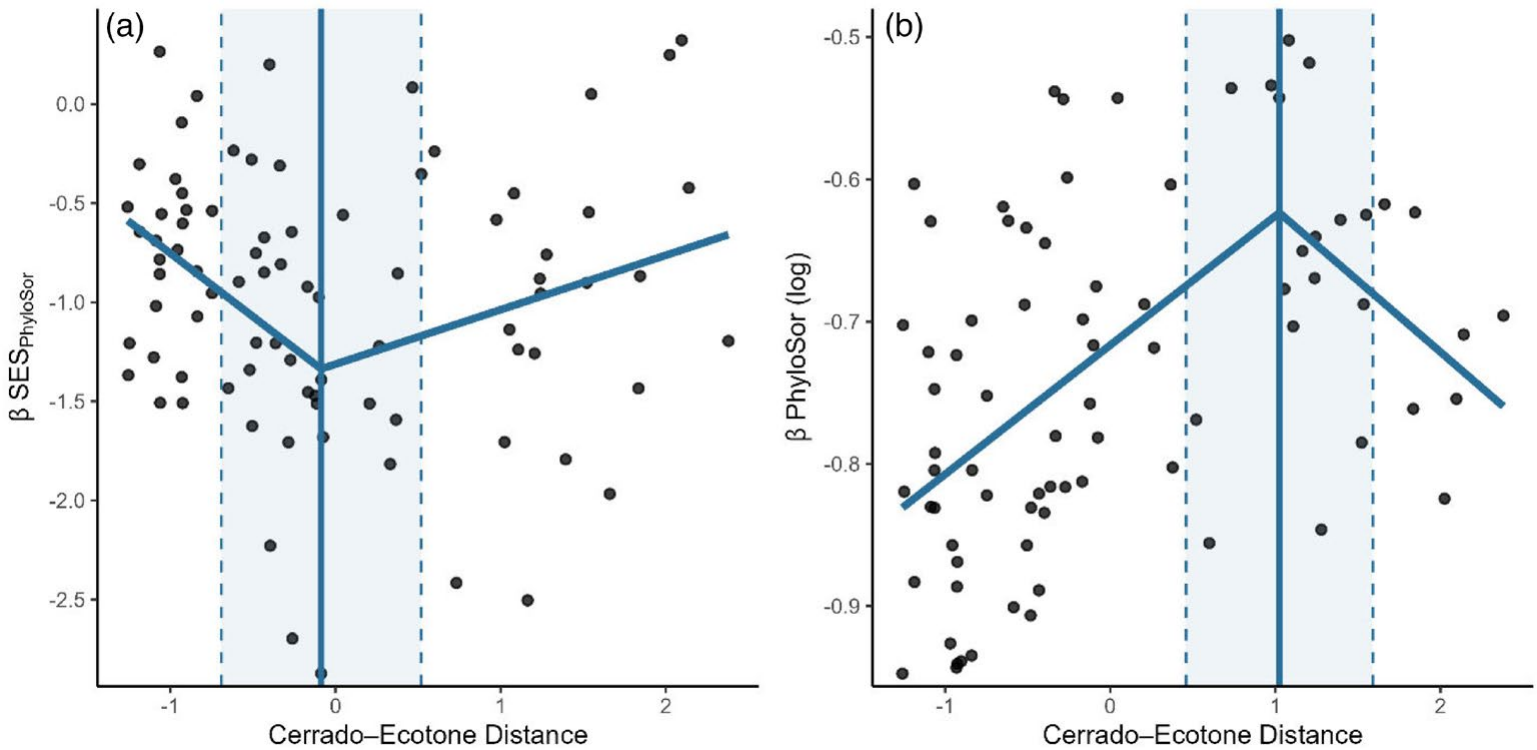
Piecewise linear regressions showing breakpoints in geographical distance (Cerrado–Ecotone) versus phylogenetic beta diversity of bat communities from the Cerrado. Significant relationships between distance from the Cerrado and abrupt change in standardised (a) SES.PhyloSor and observed (b) PhyloSor (log). Estimated breakpoints (95% CI): (a) *x* = −0.087 [−0.693, 0.519]; (b) *x* = 1.024 [0.458, 1.590]. The shaded vertical band marks the 95% CI of the breakpoint; dashed lines indicate the confidence interval bounds and the solid line the point estimate.

## 4 DISCUSSION

While there are suggestions that ecotones can serve as evolutionary drivers of novelty and diversification (Kark, 2013), our results indicate that bat community assembly in the Cerrado–Atlantic Forest ecotone is determined mainly by contemporary ecological filters. Consistent with our initial predictions, we found that environmental filtering is the predominant ecological process shaping community composition in the ecotone. Additionally, our findings highlight that ecological determinants, namely, climatic variables and vertical stratification of vegetation, are the main factors driving PBD in the ecotone. We also found distinct patterns of PBD between the two biomes, particularly with respect to the distance of the ecotone from the central regions of these biomes.

### 4.1 Community assembly process

Using the CAMI statistical framework to distinguish among processes of community assembly in the ecotone, we found that the environmental filtering model had greater statistical support than the competitive exclusion and neutral assembly models. While this result contradicts our original hypothesis, it can be explained by two main lines of evidence, both relating to the same argument but on different timescales. In a recent or shorter time scale, considering the low variation in vegetation structure on a local scale in the ecotone region (Durigan & Ratter, 2006), and assuming that the chosen functional traits accurately represent the use of environmental resources, species arriving at the ecotone from the two adjacent biomes will only be able to efficiently forage and use resources if they are generalist enough to use the different (or intermediate) ecotone vegetation structure compared with the Cerrado or Atlantic Forest. This generalist resource‐use idea is further supported by the fact that most species in the ecotone co‐occur in the Cerrado and Atlantic Forest (results 3.1). Thus, the environmental filtering process will also favour the coexistence of phylogenetically closer species with similar functional traits (Cardillo, 2011; MacArthur & Levins, 1967), in this case, mostly bats in the family Phyllostomidae. Furthermore, over a longer timescale, it is likely that different species arrived at the ecotone but were consistently filtered by the distinct environment. This has resulted in lower phylogenetic diversity compared with the central biomes, suggesting a phylogenetic clustering scenario in which environmental filtering has excluded very distinct phenotypes (Fritz & Rahbek, 2012; Mazel et al., 2016).

We expected the ecotone to exhibit a more heterogeneous vegetation landscape, reflecting characteristics of both the Cerrado and the Atlantic Forest. However, most communities in the ecotone showed greater similarity to the Cerrado vegetation cover. This implies that species adapted to the climate and vegetation of the Cerrado will most likely be able to use the ecotone environment and its resources (Clare et al., 2014); hence leading to a pattern of phylogenetic clustering, where Atlantic Forest species face difficulties in establishing resident populations and are filtered out by the environmental conditions (Meyer & Kalko, 2008). This pattern can be further reinforced by the high abundance of pioneer species, which provide sufficient resources to sustain bats at lower trophic levels but are probably insufficient to support species at higher trophic levels. Additionally, our findings highlight climatic variables and canopy height as the main factors driving PBD in the ecotone area (Farneda et al., 2015).

Although inferring community assembly processes remains a challenge in ecology, our results show that niche overlap plays a fundamental role in structuring bat communities in this ecotone region. Coevolutionary models represent an important advance in understanding how environmental filtering interacts with niche overlap (Pontarp, 2021; Ruffley et al., 2019). These models help elucidate how species‐specific functional traits facilitate or limit their coexistence, indicating that functional similarity may be a key factor in the observed phylogenetic structuring and, in the case of the Cerrado–Atlantic Forest ecotone, depicts bat flight patterns and the ability to exploit environmental resources.

### 4.2 Effect of environmental variables on phylogenetic beta diversity

Although we identified similar patterns of PBD across the three studied regions, the impact of predictor variables significantly varied among them. According to our hypothesis, variation in PBD in the bat communities in the Cerrado–Atlantic Forest ecotone region was primarily influenced by contemporary climatic factors and the vertical stratification of vegetation (Figure 5). Our results reveal that SES.PBD (Standardised Effect Size Phylogenetic Beta Diversity) increases with higher precipitation seasonality and decreases as annual precipitation and annual temperature range increase. This suggests a tendency towards phylogenetic overdispersion in areas with high seasonal rainfall, where less phylogenetically related species co‐occur. In contrast, in regions with lower rainfall and temperature range, we observed phylogenetic clustering, with evolutionarily closer species sharing the same habitat. Furthermore, PBD increases in regions with higher vegetation canopy height, indicating that the vertical structure of the environment plays a crucial role in structuring bat communities in the ecotone. These contrasting results suggest that phylogenetically distinct species are found in areas with characteristics more similar to those of the Atlantic Forest. In contrast, most evolutionarily similar species may be associated with environmental gradients more characteristic of the Cerrado vegetation.

Variation in PBD can be attributed to both environmental gradients and organismal dispersal (Graham & Fine, 2008). Previous studies suggest that low dispersal or high dispersal can result in high or low PBD, respectively (Graham & Fine, 2008; Jang et al., 2022). Furthermore, coevolutionary models that combine the environmental gradient with dispersal have found a negative relationship between dispersal and PBD (Jang et al., 2022). Although we could not directly test dispersal, our results suggest that bats' dispersal ability may have influenced variation in PBD, not just the environmental gradient alone. This consideration broadens our understanding of the factors shaping PBD in ecotonal environments, such as the Cerrado–Atlantic Forest ecotone.

### 4.3 Influence of ecotone distance on abrupt change in phylogenetic beta diversity

Our hypothesis of an abrupt change in PBD variation towards the more central regions of the biomes was partially corroborated. We observed significant abrupt changes in both SES.PBD and PBD in the central regions of the Cerrado (Figure 5a,b). In the central regions of the Atlantic Forest, there was also a change, but this variation was not related to distance from the ecotone, suggesting that other factors, possibly ecological or historical, may be influencing the structure of these communities. The contrasting patterns observed at the breakpoint of PBD and SES.PBD variation in the Cerrado are important to our understanding of how PBD is shaped in these environments. In the Cerrado, we found an increase in observed PBD along the gradient from the ecotone region to approximately 300 km, followed by a decline in the more central regions of the biome. This pattern suggests that, as communities move away from the ecotone and towards the central areas of the Cerrado, phylogenetic diversity initially increases, possibly due to the presence of more phylogenetically distinct species, until it reaches a saturation point. From this breakpoint (~300 km), the decline in observed PBD may indicate that communities in central areas are becoming more phylogenetically homogeneous, reflecting a reduction in species turnover. In contrast, the increase in SES.PBD after the breakpoint suggests that although species in the central areas of the Cerrado may be more similar to one another, they remain more phylogenetically dispersed than communities closer to the ecotone. This indicates that, despite taxonomic homogeneity, the species that remain in these central areas are less evolutionarily related, which may point to competitive exclusion or to different historical processes that favoured species with distinct evolutionary histories.

The identified breakpoints, where the distribution of bat species appears to be limited, are particularly interesting. Although 59 of the 62 species present in the ecotone also occur in the Cerrado, this abrupt change suggests that factors such as dispersal limitation (Trevelin et al., 2013) and ecological barriers may be restricting the distribution of particular species, preventing them from penetrating the central areas. This may be reinforced by selective pressures that favour species better adapted to the more arid conditions and lower canopies of the central areas of the Cerrado. Nevertheless, it is plausible that species are undergoing directional selection induced by the environment as they move along the environmental gradient, adapting to local conditions in different habitats. This idea is consistent with the environmental selection hypothesis, where selective pressures can force species to specialise in different regions along the ecotonal gradient (Jang et al., 2022). In this context, ecotone communities can act as a filter zone, where only species with particular adaptations can expand into the Cerrado.

## 5 CONCLUSION

Considering the above, the results from the eco‐evolutionary model indicate that environmental filtering plays a crucial role in organising bat communities in the Cerrado–Atlantic Forest ecotone. Environmental variables emerge as the main predictors of PBD, reflecting the interaction between species' functional traits and local environmental conditions. Our analysis revealed that the composition of communities in the ecotone reflects aspects of both the Cerrado and the Atlantic Forest, but with a predominance of functional traits more adapted to the Cerrado. The abrupt variation between PBD and SES of PBD showed distinct patterns towards the central regions of the Cerrado, indicating an increase in PBD up to a breaking point, followed by a decline in the more central communities. This pattern suggests that species replacement along the gradient is strongly influenced by environmental filtering and possibly by competitive exclusion, with species better adapted to Cerrado conditions being favoured. Thus, our results underline the importance of considering both environmental gradients and species' capacity for mobility and dispersal when modelling PBD.

## AUTHOR CONTRIBUTIONS

Daiana Cardoso Silva, Hernani Fernandes Magalhães Oliveira and Fabricius Maia Chaves Bicalho Domingos conceived the ideas and designed the methodology. Daiana Cardoso Silva and Fabricius Maia Chaves Bicalho Domingos developed the specific aims and hypotheses. Daiana Cardoso Silva and Hernani Fernandes Magalhães Oliveira collected the data. Daiana Cardoso Silva analysed the data and led the writing of the manuscript. Hernani Fernandes Magalhães Oliveira and Fabricius Maia Chaves Bicalho Domingos critically revised the manuscript. All authors contributed substantially to the drafts, approved the final version for publication, and agree to be accountable for all aspects of the work.

## CONFLICT OF INTEREST STATEMENT

The authors declare no conflict of interest.

## Supporting information


**Table S1.1.** Variance inflation factor (VIF) for the climate variables for each biome that were included in the analyses (VIF <3).
**Table S1.2**. Parameter estimates, log likelihoods and AIC scores for BM and OU trait evolution models. The OU model was always the best‐fitting model.
**Table S1.3**. List of species present in each environment and values for forearm, mass, aspect ratio (AR) and wing loading (WL). The values in each environment represent the number of communities in which the species is present. In * are the species present in the three environments, Ecotone, Cerrado and Atlantic Forest, ** are the species present in Ecotone and Cerrado, *** are the species present in the Ecotone and Atlantic Forest. Those numbered 1–3 are those present in the Cerrado, Atlantic Rainforest, and those that co‐occur in the Cerrado and Atlantic Rainforest, respectively. Missing WL and AR values were imputed using phylogenetic generalised least squares (PGLS) models with forearm length as predictor.
**Table S1.4**. Predictions from the RF community assembly model and posterior probabilities from the ABC model for all bat communities in the Cerrado–Atlantic Forest ecotone.
**Table S1.5**. Probabilities of the competitive exclusion model for the 24 bat communities of the Cerrado–Atlantic Forest ecotone region using RF and ABC, as well as the median estimates $\;t_{E}$ using ABC.
**Figure S2.1**. Vegetation coverage (%) of all communities in the Cerrado, Ecotone, and Atlantic Forest; *n* = 84, 24, 117, respectively. Medians: Cerrado 19.8%, Ecotone 14.5%, Atlantic Forest 35.1%. The ecotone is more similar to Cerrado (KS distance Eco–Cer = 0.274; Eco–AF = 0.464).
**Figure S2.2**. Error rates or proportion of incorrectly classified simulations when classifying community assembly and attribute evolution models with the 1000 random forest trees.
**Figure S2.3**. Probability distributions a priori (light grey) and posterior (dark grey) of $t_{E}$ in a model of environmental filtering and trait evolution under the OU model for bat communities from the Cerrado–Atlantic Forest ecotone. The dotted line represents the median estimate of $t_{E}$.
**Figure S2.4**. Linear regression models between the model support for competitive exclusion as predicted by RF and ABC and the median estimates $\;t_{E}$ of ABC.
**Figure S2.5**. Simple regression to indicate the general directions of the relationship between environmental variables and Cerrado phylobetadiversity metrics. Importance of variables chosen for representation were estimated by *Random Forest*. Standardised phylogenetic beta diversity in relation to precipitation of coldest quarter (a), precipitation of warmest quarter (b) and canopy height (c). Phylogenetic beta diversity observed in relation to precipitation of warmest quarter (d), precipitation of coldest quarter (e), mean temperature of wettest quarter (f) and palaeoclimate (g).
**Figure S2.6**. Simple regression to indicate the general directions of the relationship between environmental variables and Atlantic Forest phylobetadiversity metrics. Importance of variables chosen for representation were estimated by *Random Forest*. Phylogenetic beta diversity standardised in relation to elevation (a) and palaeoclimate (b). Phylogenetic beta diversity observed in relation to elevation (c), canopy height (d), temperature annual range (e) and paleoclimate (f).

## Data Availability

Data available from the Dryad Digital Repository https://doi.org/10.5061/dryad.mgqnk99d2 (Silva et al., 2026).
