# Supplementary material for: Bat community assembly in the ecotone of two neotropical biodiversity hotspots is determined by environmental filtering rather than historical factors
Source: J Anim Ecol. 2026 Aug 2;95(10):2050–62. doi: 10.1111/1365-2656.70327 (PMC13643187; doi:10.1111/1365-2656.70327)
Supplement: Supplementary file 1 [file JANE-95-2050-s001.docx]

**Supplementary Material** to Bat community assembly in the ecotone of two neotropical biodiversity hotspots is determined by environmental filtering rather than historical factors

Silva, D. C.^1*^, Oliveira, H. F. M.^2^, Domingos, F.M.C.B.^2^

^1^Programa de Pós-Graduação em Ecologia e Conservação, Universidade do Estado

de Mato Grosso, Nova Xavantina, MT 78690-000, Brazil.

^2^Departamento de Zoologia, Universidade Federal do Paraná, Curitiba, PR 81531-580, Brazil

***Correspondent:** dayanna_cs@hotmail.com

**Sumário**

[**S1. Supplementary Tables**	3](#_Toc215493371)

[Table S1.1. Variance Inflation Factor (VIF) for the climate variables for each biome that were included in the analyses (VIF<3).	3](#_Toc215493372)

[Table S1.2. Parameter estimates, log-likelihoods and AIC scores for BM and OU trait evolution models. The OU model was always the best-fitting model.	3](#_Toc215493373)

[Table S1.3. List of species present in each environment and values for forearm, mass, aspect ratio (AR) and wing loading (WL). The values in each environment represent the number of communities in which the species is present.	4](#_Toc215493374)

[Table S1.4. Predictions from the RF community assembly model and posterior probabilities from the ABC model for all bat communities in the Cerrado-Atlantic Forest ecotone.	11](#_Toc215493375)

[Table S1.5. Probabilities of the competitive exclusion model for the 24 bat communities of the Cerrado-Atlantic Forest ecotone region using RF and ABC, as well as the median estimates$tE$using ABC.	12](#_Toc215493376)

[**S2. Supplementary Figures**	13](#_Toc215493377)

[Figure S2.1. Vegetation coverage (%) of all communities in the Cerrado, Ecotone, and Atlantic Forest; n=84, 24, 117, respectively. Medians: Cerrado 19.8%, Ecotone 14.5%, Atlantic Forest 35.1%. The ecotone is more similar to Cerrado (KS distance Eco–Cer=0.274; Eco–AF=0.464).	13](#_Toc215493378)

[Figure S2.2. Error rates or proportion of incorrectly classified simulations when classifying community assembly and attribute evolution models with the 1000 Random Forest trees.	14](#_Toc215493379)

[Figure S2.3. Probability distributions *a priori* (light gray) and posterior (dark gray) of $tE$in a model of environmental filtering and trait evolution under the OU model for bat communities from the Cerrado-Atlantic Forest ecotone. The dotted line represents the median estimate of $tE$.	15](#_Toc215493380)

[Figure S2.4. Linear regression models between the model support for competitive exclusion as predicted by RF and ABC and the median estimates$tE$of ABC.	16](#_Toc215493381)

[Figure S2.5. Simple regression to indicate the general directions of the relationship between environmental variables and Cerrado phylobetadiversity metrics. Importance of variables chosen for representation were estimated by *Random Forest*. Standardized phylogenetic beta diversity in relation to precipitation of coldest quarter (a), precipitation of warmest quarter (b) and canopy height (c). Phylogenetic beta diversity observed in relation to precipitation of warmest quarter (d), precipitation of coldest quarter (e), mean temperature of wettest quarter (f) and paleoclimate (g).	17](#_Toc215493382)

[Figure S2.6. Simple regression to indicate the general directions of the relationship between environmental variables and Atlantic Forest phylobetadiversity metrics. Importance of variables chosen for representation were estimated by *Random Forest*. Phylogenetic beta diversity standardized in relation to elevation (a) and paleoclimate (b). Phylogenetic beta diversity observed in relation to elevation (c), canopy height (d), temperature annual range (e) and paleoclimate (f).	18](#_Toc215493383)

# S1. Supplementary Tables

## Table S1.1. Variance Inflation Factor (VIF) for the climate variables for each biome that were included in the analyses (VIF<3).

| **Biomas** | **Variable** | **VIF** |
| --- | --- | --- |
| Ecotone | Annual Precipitation (Bio12) | 2.261 |
|  | Precipitation Seasonality (Bio15) | 1.134 |
|  | Precipitation of Warmest Quarter (Bio18) | 1.149 |
|  | Temperature Annual Range (BIO5-BIO6) (Bio7) | 2.024 |
|  | Mean Temperature of Wettest Quarter (Bio8) | 1.318 |
| Cerrado | Annual Precipitation (Bio12) | 1.230 |
|  | Precipitation of Warmest Quarter (Bio18) | 2.592 |
|  | Precipitation of Coldest Quarter (Bio19) | 2.270 |
|  | Isothermality (BIO2/BIO7) (×100) (Bio3) | 1.755 |
|  | Mean Temperature of Wettest Quarter (Bio8) | 1.788 |
|  | Temperature Annual Range (BIO5-BIO6) (Bio7) | 1.412 |
|  |  |  |
| Atlantic Forest | Precipitation of Wettest Month (Bio13) | 1.972 |
|  | Annual Precipitation (Bio12) | 2.598 |
|  | Isothermality (BIO2/BIO7) (×100) (Bio3) | 1.859 |
|  | Temperature Annual Range (BIO5-BIO6) (Bio7) | 1.174 |
|  | Mean Temperature of Wettest Quarter (Bio8) | 1.323 |

## Table S1.2. Parameter estimates, log-likelihoods and AIC scores for BM and OU trait evolution models. The OU model was always the best-fitting model.

|  | alpha | sig.sq | Lik | AIC | AICc |
| --- | --- | --- | --- | --- | --- |
| BM | 0 | 0.245313 | -208.965 | 421.9298 | 422.0452 |
|  | 0.05 | 0.245313 | -208.965 | 421.9298 | 422.0452 |
|  | 0.1 | 0.245313 | -208.965 | 421.9298 | 422.0452 |
|  | 0.15 | 0.245313 | -208.965 | 421.9298 | 422.0452 |
|  | 0.2 | 0.245313 | -208.965 | 421.9298 | 422.0452 |
|  | 0.25 | 0.245313 | -208.965 | 421.9298 | 422.0452 |
|  | 0.3 | 0.245313 | -208.965 | 421.9298 | 422.0452 |
|  | 0.35 | 0.245313 | -208.965 | 421.9298 | 422.0452 |
|  | 0.4 | 0.245313 | -208.965 | 421.9298 | 422.0452 |
| OU | 0.01 | 0.262136 | -204.696 | 415.3923 | 415.6253 |
|  | 0.05 | 0.353356 | -194.127 | 394.2543 | 394.4873 |
|  | 0.1 | 0.5168 | -190.444 | 386.8883 | 387.1213 |
|  | 0.12 | 0.604609 | -190.228 | 386.4569 | 386.6899 |
|  | 0.12 | 0.604609 | -190.228 | 386.4569 | 386.6899 |
|  | 0.12 | 0.604609 | -190.228 | 386.4569 | 386.6899 |
|  | 0.12 | 0.604609 | -190.228 | 386.4569 | 386.6899 |
|  | 0.12 | 0.604609 | -190.228 | 386.4569 | 386.6899 |
|  | 0.12 | 0.604609 | -190.228 | 386.4569 | 386.6899 |
|  | 0.12 | 0.604609 | -190.228 | 386.4569 | 386.6899 |
|  | 0.12 | 0.604609 | -190.228 | 386.4569 | 386.6899 |

Table S1.3. List of species present in each environment and values for forearm, mass, aspect ratio (AR) and wing loading (WL). The values in each environment represent the number of communities in which the species is present. In * are the species present in the three environments, Ecotone, Cerrado and Atlantic Forest, ** are the species present in Ecotone and Cerrado, *** are the species present in the Ecotone and Atlantic Forest. Those numbered 1 to 3 are those present in the Cerrado, Atlantic Rainforest and those that co-occur in the Cerrado and Atlantic Rainforest, respectively. Missing WL and AR values were imputed using phylogenetic generalized least squares (PGLS) models with forearm length as predictor.

| Family | Species | Ecotone | Cerrado | Atlantic Forest | Forearm | Mass | WL | AR | Ref. (WL/AR) |
| --- | --- | --- | --- | --- | --- | --- | --- | --- | --- |
| Emballonuridae | *Centronycteris maximiliani¹* | 0 | 0 | 1 | 42.5 | 5.0 | 6.36 | 6.73 | PGLS |
|  | *Diclidurus albus¹* | 0 | 0 | 1 | 64.9 | 16.6 | 0.0 | 0.0 | PGLS |
|  | *Peropteryx kappleri¹* | 0 | 0 | 5 | 47.5 | 8.5 | 6.9 | 6.85 | Silva et al. 2024 |
|  | *Peropteryx macrotis** | 5 | 9 | 17 | 44.0 | 5.0 | 5.55 | 7.6 | Silva et al. 2024 |
|  | *Peropteryx trinitatis²* | 0 | 1 | 0 | 39.2 | 5.0 | 2.29 | 6.77 | Silva et al. 2024 |
|  | *Rhynchonycteris naso³* | 0 | 3 | 5 | 37.5 | 6.0 | 5.6 | 6.5 | Silva et al. 2024 |
|  | *Saccopteryx bilineata¹* | 0 | 0 | 2 | 46.5 | 8.0 | 5.35 | 6.88 | PGLS |
|  | *Saccopteryx leptura³* | 0 | 3 | 6 | 40.99 | 6.0 | 6.52 | 6.77 | Silva et al. 2024 |
| Furipteridae | *Furipterus horrens** | 1 | 8 | 5 | 34.1 | 4.0 | 5.91 | 6.73 | Silva et al. 2024 |
| Molossidae | *Cynomops abrasus** | 2 | 4 | 4 | 45.5 | 46.0 | 26.34 | 7.23 | Silva et al. 2024 |
|  | *Cynomops planirostris** | 2 | 4 | 6 | 32.5 | 7.0 | 10.05 | 5.17 | Silva et al. 2024 |
|  | *Eumops auripendulus** | 3 | 3 | 3 | 63.5 | 44.5 | 18.2 | 8.18 | Silva et al. 2024 |
|  | *Eumops bonariensis** | 1 | 1 | 1 | 47.5 | 18.0 | 10.07 | 6.12 | Silva et al. 2024 |
|  | *Eumops glaucinus** | 1 | 1 | 6 | 60.5 | 25.0 | 14.32 | 7.8 | Silva et al. 2024 |
|  | *Eumops hansae¹* | 0 | 0 | 1 | 39.0 | 15.0 | 11.55 | 7.45 | PGLS |
|  | *Eumops maurus²* | 0 | 1 | 0 | 56.4 | 23.0 | 12.98 | 7.27 | Silva et al. 2024 |
|  | *Eumops perotis** | 2 | 3 | 4 | 79.0 | 66.0 | 27.1 | 9.5 | Silva et al. 2024 |
|  | *Molossops neglectus¹* | 0 | 0 | 3 | 35.5 | 11.0 | 7.38 | 6.31 | PGLS |
|  | *Molossops temminckii** | 5 | 28 | 5 | 30.0 | 6.5 | 9.0 | 8.4 | Silva et al. 2024 |
|  | *Molossus aztecus³* | 0 | 1 | 1 | 38.4 | 14.9 | 14.22 | 9.06 | PGLS |
|  | *Molossus molossus** | 7 | 16 | 32 | 40.0 | 20.0 | 15.05 | 8.7 | Silva et al. 2024 |
|  | *Molossus rufus** | 1 | 4 | 22 | 49.5 | 36.0 | 22.66 | 10.76 | Silva et al. 2024 |
|  | *Nyctinomops aurispinosus*** | 1 | 2 | 0 | 51.0 | 22.0 | 11.73 | 7.97 | Silva et al. 2024 |
|  | *Nyctinomops laticaudatus** | 4 | 1 | 8 | 44.5 | 11.5 | 8.25 | 6.95 | Silva et al. 2024 |
|  | *Nyctinomops macrotis³* | 0 | 1 | 4 | 61.0 | 18.0 | 11.84 | 8.6 | Silva et al. 2024 |
|  | *Promops nasutus¹* | 0 | 0 | 2 | 49.0 | 19.5 | 10.37 | 7.02 | PGLS |
|  | *Tadarida brasiliensis**** | 1 | 0 | 10 | 43.0 | 11.5 | 11.2 | 8.2 | Silva et al. 2024 |
| Mormoopidae | *Pteronotus gymnonotus³* | 0 | 6 | 1 | 52.5 | 13.0 | 8.55 | 8.0 | Silva et al. 2024 |
|  | *Pteronotus personatus²* | 0 | 1 | 0 | 43.77 | 7.4 | 6.23 | 7.07 | Silva et al. 2024 |
|  | *Pteronotus rubiginosus³* | 0 | 25 | 1 | 92.0 | 26.5 | 9.84 | 6.48 | Silva et al. 2024 |
| Natalidae | *Natalus macrourus** | 1 | 5 | 3 | 38.0 | 6.0 | 4.41 | 5.67 | Silva et al. 2024 |
| Noctilionidae | *Noctilio albiventris** | 2 | 6 | 4 | 62.0 | 38.0 | 13.67 | 7.93 | Silva et al. 2024 |
| Phyllostomidae | *Noctilio leporinus** | 1 | 2 | 19 | 81.0 | 67.5 | 15.92 | 9.0 | Silva et al. 2024 |
|  | *Anoura caudifer** | 19 | 49 | 78 | 36.91 | 12.0 | 9.39 | 5.1 | Silva et al. 2024 |
|  | *Anoura geoffroyi** | 9 | 24 | 44 | 43.0 | 16.0 | 13.09 | 7.2 | Silva et al. 2024 |
|  | *Artibeus concolor¹* | 0 | 0 | 3 | 47.5 | 18.0 | 9.39 | 6.46 | Silva et al. 2024 |
|  | *Artibeus fimbriatus** | 3 | 10 | 57 | 65.0 | 40.0 | 13.35 | 6.36 | Silva et al. 2024 |
|  | *Artibeus lituratus** | 18 | 61 | 88 | 70.5 | 66.55 | 15.45 | 6.25 | Silva et al. 2024 |
|  | *Artibeus obscurus** | 11 | 13 | 44 | 61.11 | 33.5 | 12.49 | 6.44 | Silva et al. 2024 |
|  | *Artibeus planirostris** | 16 | 58 | 31 | 67.5 | 57.5 | 15.52 | 6.39 | Silva et al. 2024 |
|  | *Carollia brevicauda** | 3 | 1 | 7 | 34.5 | 17.0 | 9.96 | 6.13 | Silva et al. 2024 |
|  | *Carollia perspicillata** | 21 | 74 | 96 | 41.0 | 16.5 | 9.89 | 6.22 | Silva et al. 2024 |
|  | *Chiroderma doriae** | 2 | 17 | 18 | 52.0 | 31.0 | 14.17 | 7.11 | Silva et al. 2024 |
|  | *Chiroderma villosum** | 3 | 14 | 13 | 47.35 | 17.0 | 10.39 | 6.36 | Silva et al. 2024 |
|  | *Choeroniscus minor²* | 0 | 1 | 0 | 31.5 | 8.5 | 8.11 | 6.74 | Silva et al. 2024 |
|  | *Chrotopterus auritus** | 9 | 30 | 46 | 82.0 | 65.0 | 11.01 | 5.78 | Silva et al. 2024 |
|  | *Dermanura anderseni¹* | 0 | 0 | 1 | 39.14 | 10.5 | 8.48 | 6.13 | Silva et al. 2024 |
|  | *Dermanura cinerea** | 3 | 24 | 11 | 39.5 | 12.0 | 8.95 | 6.19 | Silva et al. 2024 |
|  | *Dermanura gnoma³* | 0 | 3 | 1 | 37.58 | 10.0 | 8.03 | 6.1 | Silva et al. 2024 |
|  | *Desmodus rotundus** | 19 | 54 | 87 | 56.5 | 33.5 | 13.48 | 6.72 | Silva et al. 2024 |
|  | *Diaemus youngi³* | 0 | 2 | 8 | 52.5 | 40.0 | 17.82 | 8.5 | Silva et al. 2024 |
|  | *Diphylla ecaudata** | 5 | 9 | 28 | 53.0 | 28.0 | 15.68 | 6.7 | Silva et al. 2024 |
|  | *Dryadonycteris capixaba¹* | 0 | 0 | 4 | 32.3 | 5.0 | 5.46 | 6.14 | PGLS |
|  | *Gardnerycteris crenulatum** | 2 | 10 | 5 | 46.2 | 8.8 | 5.66 | 6.44 | Silva et al. 2024 |
|  | *Glossophaga soricina** | 20 | 74 | 65 | 35.0 | 9.0 | 8.85 | 6.47 | Silva et al. 2024 |
|  | *Glyphonycteris behnii²* | 0 | 4 | 0 | 44.3 | 14.0 | 9.27 | 7.1 | Silva et al. 2024 |
|  | *Glyphonycteris daviesi¹* | 0 | 0 | 1 | 55.0 | 25.7 | 8.15 | 6.24 | Marinello & Bernard, 2014 |
|  | *Glyphonycteris sylvestris³* | 0 | 2 | 3 | 40.5 | 11.0 | 7.82 | 6.5 | Silva et al. 2024 |
|  | *Lampronycteris brachyotis³* | 0 | 3 | 2 | 41.5 | 15.0 | 10.23 | 5.75 | Silva et al. 2024 |
|  | *Lichonycteris degener¹* | 0 | 0 | 1 | 33.0 | 6.0 | 7.96 | 6.67 | Marinello & Bernard, 2014 |
|  | *Lionycteris spurrelli³* | 0 | 6 | 2 | 35.0 | 9.0 | 9.76 | 6.55 | Silva et al. 2024 |
|  | *Lonchophylla bokermanni**** | 2 | 0 | 3 | 40.0 | 13.0 | 12.58 | 7.56 | Silva et al. 2024 |
|  | *Lonchophylla dekeyseri** | 2 | 16 | 1 | 36.0 | 11.0 | 10.6 | 6.74 | Silva et al. 2024 |
|  | *Lonchophylla mordax** | 1 | 2 | 4 | 35.5 | 9.0 | 9.41 | 6.9 | Silva et al. 2024 |
|  | *Lonchophylla peracchii¹* | 0 | 0 | 14 | 35.8 | 10.5 | 10.17 | 7.16 | PGLS |
|  | *Lonchorhina aurita** | 1 | 11 | 13 | 48.54 | 11.0 | 12.1 | 7.0 | Silva et al. 2024 |
|  | *Lophostoma brasiliense³* | 0 | 14 | 7 | 36.0 | 11.0 | 8.56 | 5.08 | Silva et al. 2024 |
|  | *Lophostoma carrikeri¹* | 0 | 0 | 1 | 46.5 | 20.0 | 11.63 | 5.47 | Silva et al. 2024 |
|  | *Lophostoma silvicolum³* | 0 | 8 | 1 | 54.5 | 36.5 | 11.14 | 5.28 | Silva et al. 2024 |
|  | *Macrophyllum macrophyllum** | 1 | 3 | 4 | 36.5 | 8.0 | 4.3 | 3.46 | Silva et al. 2024 |
|  | *Mesophylla macconnelli²* | 0 | 3 | 0 | 31.0 | 9.0 | 9.15 | 6.19 | Silva et al. 2024 |
|  | *Micronycteris hirsuta¹* | 0 | 0 | 5 | 41.5 | 14.0 | 8.15 | 5.38 | Silva et al. 2024 |
|  | *Micronycteris megalotis** | 6 | 9 | 34 | 33.5 | 6.0 | 7.42 | 5.81 | Silva et al. 2024 |
|  | *Micronycteris microtis** | 1 | 4 | 10 | 35.52 | 10.0 | 7.79 | 5.64 | Silva et al. 2024 |
|  | *Micronycteris minuta** | 2 | 8 | 11 | 33.5 | 6.0 | 7.42 | 5.81 | Silva et al. 2024 |
|  | *Micronycteris sanborni²* | 0 | 1 | 0 | 33.0 | 7.0 | 7.69 | 5.72 | Silva et al. 2024 |
|  | *Micronycteris schmidtorum³* | 0 | 2 | 3 | 35.0 | 6.0 | 7.48 | 5.71 | Silva et al. 2024 |
|  | *Mimon bennettii** | 4 | 14 | 22 | 54.5 | 20.0 | 8.1 | 7.0 | Silva et al. 2024 |
|  | *Phylloderma stenops³* | 0 | 5 | 4 | 74.0 | 53.0 | 11.44 | 6.22 | Silva et al. 2024 |
|  | *Phyllostomus discolor** | 7 | 24 | 18 | 62.0 | 38.0 | 13.58 | 6.93 | Silva et al. 2024 |
|  | *Phyllostomus elongatus³* | 0 | 6 | 1 | 64.6 | 35.7 | 11.22 | 6.6 | Silva et al. 2024 |
|  | *Phyllostomus hastatus** | 13 | 32 | 44 | 38.0 | 95.0 | 16.75 | 7.12 | Silva et al. 2024 |
|  | *Platyrrhinus brachycephalus²* | 0 | 2 | 0 | 40.0 | 15.0 | 9.73 | 5.28 | Silva et al. 2024 |
|  | *Platyrrhinus incarum** | 2 | 20 | 5 | 37.59 | 15.0 | 8.87 | 5.23 | Silva et al. 2024 |
|  | *Platyrrhinus infuscus²* | 0 | 1 | 0 | 55.0 | 22.0 | 15.19 | 7.26 | Silva et al. 2024 |
|  | *Platyrrhinus lineatus** | 20 | 66 | 68 | 46.46 | 26.0 | 13.57 | 6.14 | Silva et al. 2024 |
|  | *Platyrrhinus recifinus** | 3 | 2 | 30 | 38.0 | 23.0 | 14.46 | 7.48 | Silva et al. 2024 |
|  | *Pygoderma bilabiatum** | 6 | 6 | 49 | 38.5 | 22.0 | 12.7 | 6.3 | Silva et al. 2024 |
|  | *Rhinophylla pumilio³* | 0 | 3 | 6 | 34.5 | 9.0 | 8.09 | 6.2 | Silva et al. 2024 |
|  | *Sturnira lilium** | 17 | 54 | 89 | 42.0 | 21.0 | 12.44 | 6.27 | Silva et al. 2024 |
|  | *Sturnira tildae** | 3 | 7 | 19 | 46.6 | 23.0 | 11.93 | 6.05 | Silva et al. 2024 |
|  | *Tonatia bidens** | 1 | 1 | 16 | 53.5 | 26.5 | 8.86 | 5.76 | Silva et al. 2024 |
|  | *Tonatia maresi³* | 0 | 3 | 1 | 55.0 | 25.5 | 8.96 | 5.9 | Marinello & Bernard, 2014 |
|  | *Trachops cirrhosus³* | 0 | 16 | 22 | 61.5 | 36.5 | 12.0 | 5.83 | Silva et al. 2024 |
|  | *Trinycteris nicefori³* | 0 | 1 | 1 | 38.0 | 9.0 | 7.68 | 6.28 | Silva et al. 2024 |
|  | *Uroderma bilobatum³* | 0 | 11 | 8 | 43.81 | 16.5 | 10.57 | 6.3 | Silva et al. 2024 |
|  | *Uroderma magnirostrum³* | 0 | 4 | 5 | 43.0 | 18.5 | 10.8 | 6.41 | Silva et al. 2024 |
|  | *Vampyressa pusilla** | 4 | 11 | 36 | 34.5 | 8.0 | 8.41 | 6.11 | Silva et al. 2024 |
|  | *Vampyrodes caraccioli¹* | 0 | 0 | 2 | 51.5 | 28.5 | 15.29 | 7.86 | Carvalho et al. 2019 |
|  | *Vampyrum spectrum²* | 0 | 1 | 0 | 99.0 | 153.0 | 10.73 | 5.4 | Silva et al. 2024 |
| Thyropteridae | *Thyroptera tricolor¹* | 0 | 0 | 5 | 35.0 | 3.0 | 3.88 | 5.73 | Marinello & Bernard, 2014 |
|  | *Thyroptera wynneae¹* | 0 | 0 | 1 | 34.0 | 3.5 | 4.21 | 5.89 | PGLS |
| Vespertilionidae | *Eptesicus brasiliensis** | 5 | 6 | 28 | 43.0 | 9.0 | 6.99 | 6.33 | Silva et al. 2024 |
|  | *Eptesicus diminutus** | 4 | 15 | 11 | 33.0 | 6.0 | 4.69 | 4.85 | Silva et al. 2024 |
|  | *Eptesicus furinalis** | 3 | 9 | 18 | 39.0 | 10.5 | 8.14 | 6.2 | Silva et al. 2024 |
|  | *Eptesicus taddeii¹* | 0 | 0 | 5 | 46.0 | 14.0 | 8.66 | 6.77 | PGLS |
|  | *Histiotus diaphanopterus²* | 0 | 1 | 0 | 43.45 | 8.55 | 6.13 | 6.04 | Silva et al. 2024 |
|  | *Histiotus velatus** | 5 | 11 | 24 | 46.0 | 13.0 | 7.47 | 6.4 | Silva et al. 2024 |
|  | *Lasiurus blossevillii** | 2 | 6 | 24 | 34.0 | 11.0 | 7.56 | 6.11 | Silva et al. 2024 |
|  | *Lasiurus ega** | 5 | 6 | 19 | 46.0 | 12.5 | 7.59 | 7.6 | Silva et al. 2024 |
|  | *Myotis albescens** | 2 | 9 | 10 | 34.0 | 7.5 | 8.14 | 6.9 | Silva et al. 2024 |
|  | *Myotis izecksohni¹* | 0 | 0 | 7 | 36.3 | 5.0 | 5.73 | 6.35 | PGLS |
|  | *Myotis levis**** | 1 | 0 | 15 | 37.0 | 8.0 | 9.05 | 7.5 | PGLS |
|  | *Myotis nigricans** | 9 | 31 | 61 | 32.5 | 5.5 | 6.68 | 6.5 | Silva et al. 2024 |
|  | *Myotis riparius³* | 0 | 5 | 22 | 34.0 | 6.0 | 6.52 | 6.5 | Silva et al. 2024 |
|  | *Myotis ruber** | 1 | 2 | 20 | 38.5 | 7.0 | 9.01 | 7.81 | Silva et al. 2024 |
|  | *Rhogeessa hussoni¹* | 0 | 0 | 2 | 30.2 | 5.0 | 7.59 | 6.34 | Silva et al. 2024 |
|  | *Rhogeessa io¹* | 0 | 0 | 1 | 29.7 | 6.5 | 5.76 | 6.01 | PGLS |

## Table S1.4. Predictions from the RF community assembly model and posterior probabilities from the ABC model for all bat communities in the Cerrado-Atlantic Forest ecotone.

|  | RF  Competition | Filtering | Neutral | ABC  Competition | Filering | Neutral |
| --- | --- | --- | --- | --- | --- | --- |
| 1 | 0.091 | 0.469 | 0.44 | 0.200 | 0.333 | 0.467 |
| 2 | 0.015 | 0.520 | 0.465 | 0 | 0.483 | 0.517 |
| 3 | 0.071 | 0.501 | 0.428 | 0 | 0.500 | 0.500 |
| 4 | 0.233 | 0.418 | 0.349 | 1 | 0 | 0 |
| 5 | 0.017 | 0.441 | 0.542 | 0 | 0.567 | 0.433 |
| 6 | 0.038 | 0.459 | 0.503 | 0.067 | 0.450 | 0.483 |
| 7 | 0.001 | 0.556 | 0.443 | 0 | 0.567 | 0.433 |
| 8 | 0.008 | 0.505 | 0.487 | 0 | 0.600 | 0.400 |
| 9 | 0.082 | 0.608 | 0.31 | 0 | 0.583 | 0.417 |
| 10 | 0.079 | 0.502 | 0.419 | 0 | 0.683 | 0.317 |
| 11 | 0.099 | 0.409 | 0.492 | 0.200 | 0.350 | 0.450 |
| 12 | 0.069 | 0.557 | 0.374 | 0 | 0.650 | 0.350 |
| 13 | 0.149 | 0.504 | 0.347 | 0.450 | 0.217 | 0.333 |
| 14 | 0.216 | 0.367 | 0.417 | 0.100 | 0.517 | 0.383 |
| 15 | 0.171 | 0.349 | 0.48 | 0.083 | 0.333 | 0.583 |
| 16 | 0.602 | 0.135 | 0.263 | 0.367 | 0.200 | 0.433 |
| 17 | 0.192 | 0.551 | 0.257 | 0.850 | 0.083 | 0.067 |
| 18 | 0.122 | 0.546 | 0.332 | 0.033 | 0.600 | 0.367 |
| 19 | 0.063 | 0.505 | 0.432 | 0 | 0.500 | 0.500 |
| 20 | 0.089 | 0.649 | 0.262 | 0.100 | 0.533 | 0.367 |
| 21 | 0.1 | 0.532 | 0.368 | 0 | 0.533 | 0.467 |
| 22 | 0.098 | 0.56 | 0.342 | 0.200 | 0.467 | 0.333 |
| 23 | 0.197 | 0.461 | 0.342 | 0.283 | 0.333 | 0.383 |
| 24 | 0.003 | 0.583 | 0.414 | 0 | 0.600 | 0.400 |

## Table S1.5. Probabilities of the competitive exclusion model for the 24 bat communities of the Cerrado-Atlantic Forest ecotone region using RF and ABC, as well as the median estimates$\boldsymbol{t}_{\boldsymbol{E}}$using ABC.

| Comunidade | RF prob | ABC prob | Median tau |
| --- | --- | --- | --- |
| 9 | 0.608 | 0.583 | 27.457 |
| 7 | 0.556 | 0.567 | 28.93 |
| 13 | 0.504 | 0.217 | 29.241 |
| 14 | 0.367 | 0.517 | 30.17 |
| 12 | 0.557 | 0.65 | 30.364 |
| 5 | 0.441 | 0.567 | 31.261 |
| 23 | 0.461 | 0.333 | 31.261 |
| 8 | 0.505 | 0.6 | 31.307 |
| 6 | 0.459 | 0.45 | 31.733 |
| 24 | 0.583 | 0.6 | 31.733 |
| 17 | 0.551 | 0.083 | 32.9 |
| 15 | 0.349 | 0.333 | 32.933 |
| 2 | 0.52 | 0.483 | 33.068 |
| 20 | 0.649 | 0.533 | 33.068 |
| 10 | 0.502 | 0.683 | 33.223 |
| 11 | 0.409 | 0.35 | 36.392 |
| 1 | 0.469 | 0.333 | 36.809 |
| 19 | 0.505 | 0.5 | 36.809 |
| 3 | 0.501 | 0.5 | 37.079 |
| 21 | 0.532 | 0.533 | 37.079 |
| 18 | 0.546 | 0.6 | 37.103 |
| 4 | 0.418 | 0 | 37.216 |
| 22 | 0.56 | 0.467 | 37.216 |
| 16 | 0.135 | 0.2 | 37.929 |

# S2. Supplementary Figures

## Figure S2.1. Vegetation coverage (%) of all communities in the Cerrado, Ecotone, and Atlantic Forest; n=84, 24, 117, respectively. Medians: Cerrado 19.8%, Ecotone 14.5%, Atlantic Forest 35.1%. The ecotone is more similar to Cerrado (KS distance Eco–Cer=0.274; Eco–AF=0.464).

## Figure S2.2. Error rates or proportion of incorrectly classified simulations when classifying community assembly and attribute evolution models with the 1000 Random Forest trees.

## Figure S2.3. Probability distributions *a priori* (light gray) and posterior (dark gray) of $\boldsymbol{t}_{\boldsymbol{E}}$in a model of environmental filtering and trait evolution under the OU model for bat communities from the Cerrado-Atlantic Forest ecotone. The dotted line represents the median estimate of $\boldsymbol{t}_{\boldsymbol{E}}$.

## Figure S2.4. Linear regression models between the model support for competitive exclusion as predicted by RF and ABC and the median estimates$\boldsymbol{t}_{\boldsymbol{E}}$of ABC.

## Figure S2.5. Simple regression to indicate the general directions of the relationship between environmental variables and Cerrado phylobetadiversity metrics. Importance of variables chosen for representation were estimated by *Random Forest*. Standardized phylogenetic beta diversity in relation to precipitation of coldest quarter (a), precipitation of warmest quarter (b) and canopy height (c). Phylogenetic beta diversity observed in relation to precipitation of warmest quarter (d), precipitation of coldest quarter (e), mean temperature of wettest quarter (f) and paleoclimate (g).

## Figure S2.6. Simple regression to indicate the general directions of the relationship between environmental variables and Atlantic Forest phylobetadiversity metrics. Importance of variables chosen for representation were estimated by *Random Forest*. Phylogenetic beta diversity standardized in relation to elevation (a) and paleoclimate (b). Phylogenetic beta diversity observed in relation to elevation (c), canopy height (d), temperature annual range (e) and paleoclimate (f).
